# Deep learning approach for chemistry and processing history prediction from materials microstructure

**DOI:** 10.1038/s41598-022-08484-7

**Published:** 2022-03-16

**Authors:** Amir Abbas Kazemzadeh Farizhandi, Omar Betancourt, Mahmood Mamivand

**Affiliations:** 1 Computer Science Department, Boise State University, Boise, ID 83702 USA; 2 Department of Mechanical Engineering, University of California-Berkeley, Berkeley, CA 94720 USA; 3 Department of Mechanical and Biomedical Engineering, Boise State University, Boise, ID 83706 USA

**Keywords:** Computational methods, Computer science, Computational science, Materials science

## Abstract

Finding the chemical composition and processing history from a microstructure morphology for heterogeneous materials is desired in many applications. While the simulation methods based on physical concepts such as the phase-field method can predict the spatio-temporal evolution of the materials’ microstructure, they are not efficient techniques for predicting processing and chemistry if a specific morphology is desired. In this study, we propose a framework based on a deep learning approach that enables us to predict the chemistry and processing history just by reading the morphological distribution of one element. As a case study, we used a dataset from spinodal decomposition simulation of Fe–Cr–Co alloy created by the phase-field method. The mixed dataset, which includes both images, i.e., the morphology of Fe distribution, and continuous data, i.e., the Fe minimum and maximum concentration in the microstructures, are used as input data, and the spinodal temperature and initial chemical composition are utilized as the output data to train the proposed deep neural network. The proposed convolutional layers were compared with pretrained EfficientNet convolutional layers as transfer learning in microstructure feature extraction. The results show that the trained shallow network is effective for chemistry prediction. However, accurate prediction of processing temperature requires more complex feature extraction from the morphology of the microstructure. We benchmarked the model predictive accuracy for real alloy systems with a Fe–Cr–Co transmission electron microscopy micrograph. The predicted chemistry and heat treatment temperature were in good agreement with the ground truth.

## Introduction

Heterogeneous materials are widely used in various industries such as aerospace, automotive, and construction. These materials’ properties greatly depend on their microstructure, which is a function of the chemical composition and operational process of materials production. To accelerate the novel materials design process, the construction of process-structure–property (PSP) linkages is necessary. Establishing PSP linkages with sole experiments is not practical as the process is costly and time-consuming. Therefore, computational methods are used to study the structure of materials and their properties. A basic assumption for computational modeling of materials is that they are periodic on the microscopic scale and can be approximated by representative elements (RVE)^1^. Finding the effects of process conditions and the chemical composition on the characteristics of the RVE, such as volume fraction, microstructure, grain size, and consequently, the materials’ properties, will lead to the development of PSP linkages. In the past two decades, the phase-field (PF) method has been increasingly used as a robust method for studying the spatio-temporal evolution of the materials’ microstructure and physical properties^2^. It has been widely used to simulate different evolutionary phenomena, including grain growth and coarsening^3^, solidification^4^, thin-film deposition^5^, dislocation dynamics^6^, vesicle formation in biological membranes^7^, and crack propagation^8^. PF models solve a system of partial differential equations (PDEs) for a set of continuous variables of the processes. However, solving high fidelity PF equations is inherently computationally expensive because it requires solving several coupled PDEs simultaneously^9^. Therefore, PSP construction, particularly for complex materials, only based on the PF method is inefficient. To address this challenge, machine learning (ML) methods have recently been proposed as an alternative for creating these linkages based on the limited experimental/simulation data or both^10^.

Artificial intelligence (AI), ML, and data science are beneficial in speeding up and simplifying the process of discovering new materials^11^. In recent years, using data science in various fields of materials science has increased significantly^12–17^. For instance, data science is applied to help density functional theory calculations to establish a relationship between atoms’ interaction with the properties of materials based on quantum mechanics^18–21^. AI is also utilized to establish PSP linkages in the context of materials mechanics. In this case, ML can be used to design new materials with desired properties or employed to optimize the production process of the existing materials for properties improvement. Through data science, researchers will be able to examine the complex and nonlinear behavior of a materials production process that directly affects the materials’ properties^22^. Many studies have focused on solving cause-effect design, i.e., finding the material properties from the microstructure or processing history. These studies have attempted to predict the structure of the materials from processing parameters or materials properties from microstructure and processing history^10,12,23–30^. A less addressed but essential problem is a goal-driven design that tries to find the processing history of the materials from their microstructures. In these cases, the optimal microstructure that provides the optimal properties is known, e.g., via physics-based models, and it is desirable to find the chemistry and processing routes that would lead to the desirable microstructure.

The use of microstructure images in ML modeling is challenging. The microstructure quantification has been reported as the central nucleus in PSP linkages construction^24^. Microstructure quantification is important from two perspectives. First, it can increase the accuracy of the developed data-driven model. Second, an in-depth understanding of the microstructures can improve the comprehension of the effects of process variables and chemical composition on the properties of materials^24^. In recent years, deep learning (DL) methods have been successfully used in other fields, such as computer vision. Their limited applications in materials science have also proven them as reliable and promising methods^25^. The main advantages of DL methods are their simplicity, flexibility, and applicability for all types of microstructures. Furthermore, DL has been broadly applied in material science to improve the targeted properties^21,26–33^. One form of DL models that has been extensively used for feature extraction in various applications such as image, video, voice, and natural language processing is Convolutional Neural Networks (CNN)^34–37^. In materials science, CNN has been used for various image-related problems. Cang et al. used CNN to achieve a 1000-fold dimension reduction from the microstructure space^38^. DeCost et al.^39^ applied CNN for microstructure segmentation. Xie and Grossman^40^ used CNN to quantify the crystal graphs to predict the material properties. Their developed framework was able to predict eight different material properties such as formation energy, bandgap, and shear moduli with high accuracy. CNN has also been employed to index the electron backscatter diffraction patterns and determine the crystalline materials’ crystal orientation^41^. The stiffness in two-phase composites has been predicted successfully by the deep learning approach, including convolutional and fully-connected layers^42^. In a comparative study, the CNN and the materials knowledge systems (MKS), proposed in the Kalidindi group based on the idea of using the *n*-point correlation method for microstructures quantification^43–45^, were used for microstructure quantification and then, the produced data were employed to predict the strain in the microstructural volume elements. The comparison showed that the extracted features by CNN could provide more accurate predictions^46^. Cecen et al.^47^ proposed CNN to find the salient features of a collection of 5900 microstructures. The results showed that the obtained features from CNN could predict the properties more accurately than the 2-point correlation, while the computation cost was also significantly reduced. Comparing DL approaches, including CNN, with the MKS method, single-agent, and multi-agent methods shows that DL always performs more accurately^46,48,49^. Zhao et al. utilized the electronic charge density (ECD) as a generic unified 3D descriptor for elasticity prediction. The results showed a better prediction power for bulk modulus than shear modulus^50^. CNN has also been applied for finding universal 3D voxel descriptors to predict the target properties of solid-state material^51^. The introduced descriptors outperformed the other descriptors in the prediction of Hartree energies for solid-state materials.

Training a deep CNN usually requires an extensive training dataset that is not always available in many applications. Therefore, a transfer learning method that uses a pretrained network can be applied for new applications. In transfer learning, all or a part of the pretrained networks such as VGG16, VGG19^52^, Xception^53^, ResNet^54^, and Inception^55^, which were trained by computer vision research community with lots of open source image datasets such as ImageNet, MS, CoCo, and Pascal, can be used for the desired application. In particular, in materials science which generally the image-based data are not greatly abundant, transfer learning could be beneficial. DeCost et al.^56^ adopted VGG16 to classify the microstructures based on their annealing conditions. Ling et al.^12^ applied VGG16 to extract the feature from scanning electron microscope (SEM) images and classify them. Lubbers et al.^57^ used the VGG 19 pretrained model to identify the physical meaningful descriptors in microstructures. Li et al.^58^ proposed a framework based on VGG19 for microstructure reconstruction and structure–property predictions. The pretrained VGG19 network was also utilized to reconstruct the 3D microstructures from 2D microstructures by Bostanabad^59^.

Review provided above shows that the majority of the ML-microstructure related works in the materials science community were primarily focused on using ML techniques for microstructure classification^60–62^, recognition^63^, microstructure reconstruction^58,59^, or as a feature-engineering-free framework to connect microstructure to the properties of the materials^42,64,65^. However, the process and chemistry prediction from a microstructure morphology image have received limited attention. This is a critical knowledge gap to address specifically for the problems in them the ideal microstructure or morphology with the specific chemistry associated with the morphology domains are known, but the chemistry and processing which would lead to that ideal morphology is unknown. The problem becomes much more challenging for multicomponent alloys with complex processing steps. Recently, Kautz et al.^65^ have used the CNN for microstructure classification and segmentation on Uranium alloyed with 10 wt% molybdenum (U-10Mo). They used the segmentation algorithm to calculate the area fraction of the lamellar transformation products of α-U + γ-UMo, and by feeding the total area fraction into the Johnson–Mehl–Avrami-Kolmogorov equation, they were able to predict the annealing parameters, i.e., time and temperature. However, Kautz’s et al.^65^ work for aging time prediction did not consider the morphology and particle distribution, and also, no chemistry was involved in the model. To address the knowledge gap, in this work we develop a mixed-data deep neural network that is capable to predict the chemistry and processing history of a micrograph. The model alloy used in this work is Fe–Cr–Co permanent magnets. These alloys experience spinodal decomposition at temperatures around 853 – 963 K. We use the PF method to create the training and test dataset for the DL network. CNN will quantify the produced microstructures by the PF method, then the salient features will be used by another deep neural network to predict the temperature and chemical composition.

## Methods

### Phase-field modeling

With the enormous increase in computational power and advances in numerical methods, the PF approach has become a powerful tool for quantitative modeling of microstructures' temporal and spatial evolution. Some applications of this method include modeling materials undergoing martensitic transformation^66^, crack propagation^67^, grain growth^68^, and materials microstructure prediction for optimization of their properties^69^.

The PF method eliminates the need for the system to track each moving boundary by having the interfaces to be of finite width where they gradually transform from one composition or phase to another^2^. This essentially causes the system to be modeled as a diffusivity problem, which can be solved by using the continuum nonlinear PDEs. There are two main PF PDEs for representing the evolution of various PF variables. One being the Allen–Cahn equation^70^ for solving non-conserved order parameters (e.g., phase regions and grains), and the other one being the Cahn–Hilliard equation^71^ for solving conserved order parameters (e.g., concentrations).

Since the diffusion of constituent elements controls the process of phase separation, we only need to track the conserved variables, i.e., Fe, Cr, and Co concentration, during isothermal spinodal phase decomposition. Thus, our model will be governed by Cahn–Hilliard equations. The PF model in this work is primarily adopted from^72^. For the spinodal decomposition of the Fe–Cr–Co ternary system, the Cahn–Hilliard equations are,

1 $$ \frac{{\partial c_{Cr} }}{\partial t} = \nabla \cdot M_{Cr,Cr} \nabla \frac{{\delta F_{tot} }}{{\delta c_{Cr} }} + \nabla \cdot M_{Cr,Co} \nabla \frac{{\delta F_{tot} }}{{\delta c_{Co} }}, $$

2 $$ \frac{{\partial c_{Co} }}{\partial t} = \nabla \cdot M_{Co,Cr} \nabla \frac{{\delta F_{tot} }}{{\delta c_{Cr} }} + \nabla \cdot M_{Co,Co} \nabla \frac{{\delta F_{tot} }}{{\delta c_{Co} }}. $$

The microstructure evolution is primarily driven by the minimization of the total free energy *F*_*tot*_ of the system. The free energy functional, using *N* conserved variables *c*_*i*_ at the location $\vec{r}$ is described by:

3 $$ F_{tot} = \mathop \int \limits_{{\vec{r}}} \left[ {f_{loc} \left( {c_{1} , \ldots ,c_{N} , T} \right) + f_{gr} \left( {c_{1} , \ldots ,c_{N} } \right)} \right]d\vec{r} + E_{el} $$

In this model, *N* = 3 conserved variables are *c*_*Fe*_, *c*_*Cr*_, and *c*_*Co,*_ and they denote the composition of Fe, Cr, and Co, respectively. *f*_*gr*_ is the gradient energy density and is described by

4 $$ f_{gr} = \frac{\kappa }{2}\mathop \sum \limits_{i}^{N} \left| {\nabla c_{i} } \right|^{2} , $$

where *κ*_*i*_ is the gradient energy coefficient. In this case, *κ* is considered a constant value. *f*_*loc*_ is the local Gibbs free energy density as a function of all concentrations, *c*_*i,*_ and temperature, *T*. For this work, we will model the body-centered cubic phase of Fe–Cr–Co, where the Gibbs free energy of the system is described as^72^,

5 $$ f_{loc} = f_{Fe}^{0} c_{Fe} + f_{Cr}^{0} c_{Cr} + { }f_{Co}^{0} c_{Co} + RT\left( {c_{Fe} lnc_{Fe} + c_{Cr} lnc_{Cr} + c_{Co} lnc_{Co} } \right) + f^{E} + f^{mg} , $$

where *f*_*i*_^0^ is the Gibbs free energy of the pure element *i* and *f*^*E*^ is the excess free energy defined by

6 $$ f^{E} = L_{Fe,Cr} c_{Fe} c_{Cr} + L_{Fe,Co} c_{Fe} c_{Co} + L_{Cr,Co} c_{Cr} c_{Co} , $$

where *L*_*Fe,Cr*_, *L*_*Fe,Co*_, and *L*_*Cr,Co*_ are interaction parameters. *f*^*mg*^ is the magnetic energy contribution and can be expressed as

7 $$ f^{mg} = RTln\left( {{\upbeta } + 1} \right)f\left( \tau \right), $$

where β is the atomic magnetic moment, *f(τ)* is a function of *τ ≡ T/T*_*C*_*. T*_*C*_ is the Curie temperature. *E*_*el*_ in Eq. (3) is the elastic strain energy added to the system and is expressed as

8 $$ E_{el} = \frac{1}{2}\mathop \int \limits_{{\vec{r}}} C_{ijkl} \varepsilon_{ij}^{el} \left( {\vec{r},t} \right)\varepsilon_{kl}^{el} \left( {\vec{r},t} \right)d\vec{r}, $$

9 $$ \varepsilon_{ij}^{el} \left( {\vec{r},t} \right) = \varepsilon_{ij}^{c} \left( {\vec{r},t} \right) - \varepsilon_{ij}^{0} \left( {\vec{r},t} \right), $$

where $\varepsilon_{ij}^{el} \left( {\vec{r},t} \right)$ is the elastic strain and $C_{ijkl}$ are the elastic coefficients of the stiffness tensor. $\varepsilon_{ij}^{0} \left( {\vec{r},t} \right)$ is the eigen-strain and is expressed by

10 $$ \varepsilon_{ij}^{0} \left( {\vec{r},t} \right) = \left[ {\varepsilon_{Cr} \left( {c_{Cr} \left( {\vec{r},t} \right) - c_{Cr}^{0} } \right) + \varepsilon_{Co} \left( {c_{Co} \left( {\vec{r},t} \right) - c_{Co}^{0} } \right)} \right]\delta_{ij} , $$

where *ε*_*Cr*_ and *ε*_*Co*_ are lattice mismatches between Cr with Fe and Co with Fe, respectively. $c_{Cr}^{0}$ and $c_{Co}^{0}$ are the initial concentrations of Cr and Co, respectively and *δ*_*ij*_ is the Kronecker delta. The constrained strain, $\varepsilon_{ij}^{c} \left( {\vec{r},t} \right)$, is solved using the finite element method.

*M*_*ij*_ in Eq. (2) are Onsager coefficients and are scalar mobilities from the coupled system involving the concentrations. They can be determined by^72^,

11 $$ M_{Cr,Cr} = \left[ {c_{Fe} c_{Cr} M_{Fe} + \left( {1 - c_{cr} } \right)^{2} M_{Cr} + c_{Cr} c_{Co} M_{Co} } \right]\frac{{c_{Cr} }}{RT}, $$

12 $$ M_{Co,Co} = \left[ {c_{Fe} c_{Co} M_{Fe} + c_{Cr} c_{Co} M_{Cr} + \left( {1 - c_{Co} } \right)^{2} M_{Co} } \right]\frac{{c_{Co} }}{RT} $$

13 $$ M_{Cr,Co} = M_{Co,Cr} = \left[ {c_{Fe} M_{Fe} - \left( {1 - c_{cr} } \right)M_{Cr} - \left( {1 - c_{Co} } \right)M_{Co} } \right]\frac{{c_{Cr} c_{Co} }}{RT} $$

The mobility *M*_*i*_ of each element *i* is determined by

14 $$ M_{i} = D_{i}^{0} \exp \left( { - \frac{{Q_{i} }}{{k_{B} T}}} \right) $$

where $D_{i}^{0}$ is the self-diffusion coefficient and *Q*_*i*_ is the diffusion activation energy.

The Fe–Cr–Co evolutionary PDEs were solved using the Multiphysics Object-Oriented Simulation Environment (MOOSE) framework^73^. MOOSE is an open-source, highly parallel, finite element package developed by Idaho National Laboratory in which we took advantage of their modular structure to build our PF simulations. Using MOOSE’s prebuilt series of weak form residuals of the Cahn–Hilliard equations, we solved the coupled Cahn–Hilliard equations with the input parameters from Table S1 in Supplementary Materials.

### Training and test dataset

Since the compositions are subject to the constraint that they must sum to one, the dataset was produced based on the mixture design as a design of experiments method^74^. The Simplex-Lattice^75^ designs were adopted to provide the data for simulation. The simulation variables and their range of values are given in Table 1. The simulations were run on Boise State University R2 cluster computers^76^ using the MOOSE framework^73^.

**Table 1:** Simulation variables and their range of values for database generation.

Simulation variable	Range of values	Grid
Temperature (K)	853–963	10
Chromium composition	0.05–0.9	0.05
Cobalt composition	0.05–0.9	0.05

After running the simulations, the microstructures were collected from the results showing the phase separation. The extracted microstructures for Fe, i.e., the morphology of Fe distribution, from the PF simulations, along with the minimum and maximum compositions of Fe in each microstructure, are utilized as the inputs to predict spinodal temperature, Cr, and Co compositions as processing history parameters. Indeed, the input data is a mixed dataset combined of microstructures, as image data, and Fe composition, as numerical or continuous data. Since these values constitute different data types, the machine learning model must be able to ingest the mixed data. In general, handling the mixed data is challenging because each data type may require separate preprocessing steps, including scaling, normalization, and feature engineering^77^.

### Deep learning methodology

Deep learning (DL), as an artificial intelligence (AI) tool, is usually used for image and natural language processing as well as object and speech recognition based on human brain mimicking^36,78^. Indeed, DL is a deep neural network that can be applied for supervised, e.g., classification and regression tasks, and unsupervised, e.g., clustering, learning. In this work, since we have two different data types as input, two various networks are needed for data processing. The numerical data is fed into fully-connected layers while image features are extracted through the convolutional layers. For images involving a large number of pixel values, it is often not feasible to directly utilize all the pixel values for fully-connected layers because it can cause overfitting, increased complexity, and difficulty in model convergence. Hence, convolutional layers are applied to reduce the dimensionality of the image data by finding the image features^61,79^.

#### Fully-connected layers

Fully-connected layers are hidden layers consist of hidden neurons and activation function^80^. The number of hidden neurons is usually selected based on trial and error. The neural networks can predict complex nonlinear behaviors of systems through activation functions. Any nonlinear function that is differentiable can be used as an activation function. However, there are some activation functions such as rectified linear (ReLU), leaky rectified linear, hyperbolic tangent (Tanh), sigmoid, Swish, and softmax that have been successfully used in different applications in neural networks^81^. In particular, ReLU (f(x) = max (0, x)) and Swish (f(x) = x sigmoid(x)) activation functions have been recommended for hidden layers in deep neural networks^82^.

#### Convolutional neural networks

A convolutional neural network (CNN) is a deep network that is applied for image processing and computer vision tasks. For the first time, LeCun et al. proposed using CNN in image recognition^83^. CNN, like other deep neural networks, consists of input, output, and hidden layers. But the main difference lies in the use of hidden layers consisting of convolutional, pooling, and fully-connected layers that follow each other. Several convolutional and pooling layers can be designed in the CNN architectures.

**Figure 1:**
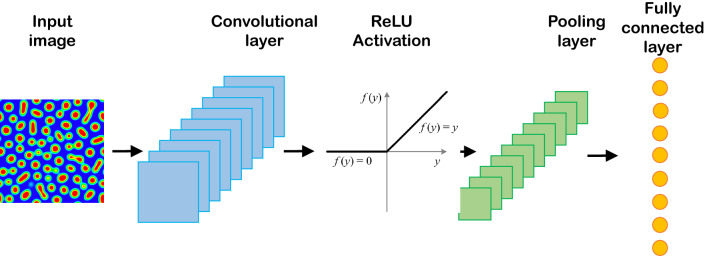
Schematic of a typical convolutional neural network.

Convolutional layers can extract the salient features of images without losing the information. At the same time, the dimensionality of the generated data gets reduced and then fed as input to the fully-connected layer. Two significant advantages of CNN are parameter sharing and sparsity of the connections. A schematic diagram for CNN is given in Fig. 1. The convolutional layer consists of filters that pass over the image and scanning the pixel values to make a feature map. The produced map proceeds through the activation function to add nonlinearity property. The pooling layer involves a pooling operation, e.g., maximum or average, which acts as a filter on the feature map. The pooling layer reduces the size of the feature map by pooling operation. Different combinations of convolutional and pooling layers are usually used in various CNN architectures. Finally, the fully-connected layers are added to train on image extracted features for a particular task such as classification or regression.

Similar to other neural networks, a cost function is used to train a CNN and update the weights and biases by backpropagation. There are many hyperparameters such as the number of filters, size of filters, regularization values, dropout values, optimizer parameters, initial weights, and biases that must be initialized before training. Training a CNN usually needs an extensive training dataset that is not always available for all applications. In this situation, transfer learning can be helpful in developing a CNN. In transfer learning, all or part of a pretrained network like VGG16, VGG19^52^, Xception^53^, ResNet^54^, and Inception^55^, which were trained by computer vision research community with lots of open source image datasets such as ImageNet, MS, CoCo, and Pascal, can be used for the desired application. The state-of-the-art pretrained network is EfficientNet which was proposed by Tan and Le^84^. This method is based on the idea that scaling up the CNN can increase its accuracy^85^. Since there was no complete understanding of the effect of network enlargement on the accuracy, Tan and Le proposed a systematic approach for scaling up the CNNs. There are different ways to scale up the CNNs by their depth^85^, width^86^, and resolution^87^. Tan and Le proposed to scale up all the depth, width, and resolution factors for the CNN with fixed scaling coefficients^84^. The results demonstrated that their proposed network, EfficientNet-B7, had better accuracy than the best-existing networks while uses 8.4 times fewer parameters and performs 6.1 times faster. In addition, they provided other EfficientNet-B0 to -B6, which can overcome the models with the corresponding scale such as ResNet-152^85^ and AmoebaNet-C^88^ in terms of accuracy with much fewer parameters. Due to the outstanding performance of EfficientNet, although it is trained based on the ImageNet dataset which is completely different from materials microstructures, it seems the EfficientNets convolutional layers have the potential to extract the features of images from other sources like materials microstructures.

#### Proposed model

**Figure 2:**
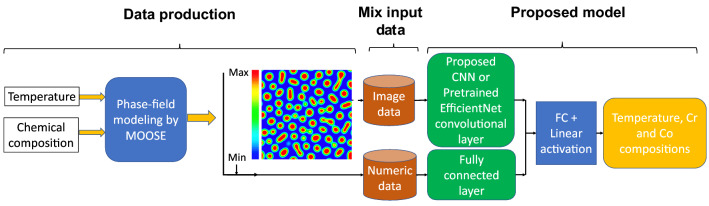
The flowchart of the developed model for chemistry and processing history prediction from microstructure images (*FC* fully-connected layer).

The training and test datasets are produced using the PF method. In this work, two different algorithms, including CNN and transfer learning, were proposed to extract the salient features of the microstructure morphologies. We applied a proposed CNN (Fig. S1) or part of pretrained EfficienctNet B-6 and B-7 convolutional layers (Fig. 2) to find the features of the microstructures. The architecture of the proposed CNN was found by testing different combinations of convolutional layers and their parameters based on the best accuracy. In the transfer learning part, different layers of the pretrained convolutional layers were tested to find the best convolutional layers for feature extraction.

On the other hand, the minimum and maximum Fe composition in the microstructure, as numerical data, is fed into the fully-connected layers. The extracted features from microstructures and the output of the fully-connected layers are combined to feed other fully-connected layers to predict the processing temperature and initial Cr and Co compositions. Different hyperparameters such as network architecture, cost function, and optimizer are tested to find the model with the highest accuracy. The model specifications, compilations (here loss function, optimizer, and metrics), and cross-validation parameters are listed in Table 2.

**Table 2:** Parameters selected for model specification, compilation, and cross-validation.

	Parameter	Selected value or option
Model specification	Learning Rate	1.00E − 0.3
	Body activation	Swish, ReLU
	Output activation	Linear
	Input dimension	(224, 224, 1)
	Output dimension	(3)
Compilation	Loss	Mean absolute percentage error
	Optimizer	Adam
	Metric	Root mean square error (RMSE), R squared
Cross-validation	Fold	5
	Training data	80%
	Testing data	20%
	Batch size	8
	Epochs	750

## Results and discussion

### Phase-field modeling and dataset generation

**Figure 3:**
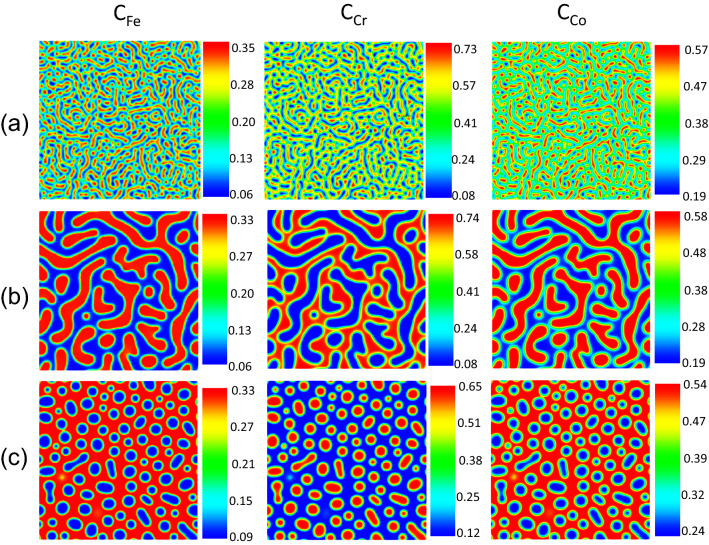
Fe–Cr–Co alloys microstructure generated by the phase-field method for: (**a**) Fe-20%, Cr-40%, Co-40% at 873 K, (**b**) Fe-20%, Cr-40%, Co-40% at 963 K, (**c**) Fe-25%, Cr-30%, Co-45% at 933 K. (Composition are in atomic percent).

Different microstructures are produced by PF modeling for different chemical compositions and temperatures. The chemical compositions and temperature were designed based on the design of experiment method. Since the chemical compositions are subject to the constraint that they must sum to one, the Simplex-Lattice design as a standard mixture design was adopted to produce the samples. In this regard, the compositions start from 0.05 and increase to 0.90 at 0.05 intervals, and the temperature rises from 853 to 963 K at 10 K increment, see Table 1. Therefore, 2053 different samples were simulated by the PF method, and the microstructures were constructed for different chemical compositions and temperatures. All the proposed operating conditions were simulated for the 100 h spinodal decomposition process. Figure 3 depicts three sample results of the PF simulation. The MOOSE-generated data can be presented in different color formats. In most transmission electron microscopy (TEM) images in literature, the Fe-rich and Cr-rich phases have been shown by bright and dark contrasts, respectively. We followed the same coloring for the extracted microstructures from the MOOSE. The Chigger python library in MOOSE has been used for microstructures extraction.

**Figure 4:**
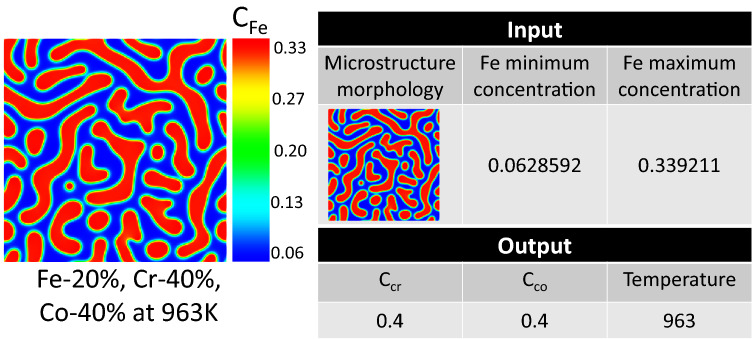
A sample workflow of dataset construction.

Since decomposition does not occur in all the proposed operating conditions and chemistries, the microstructures showing the 0.05 difference in Fe composition between Cr-rich and Fe-rich phases were considered spinodally decomposed results. Hence, 454 samples in which decomposition has taken place are used to create the database. 80% of 454 samples were used for training and 20% for testing. The training was validated by fivefold cross-validation. The Fe-based composition microstructure morphologies, as well as minimum and maximum of Fe compositions in the microstructure along with corresponding chemical compositions and temperatures, form the dataset. A sample workflow on the dataset construction is given in Fig. 4.

### Convolutional layers for feature extraction

**Figure 5:**
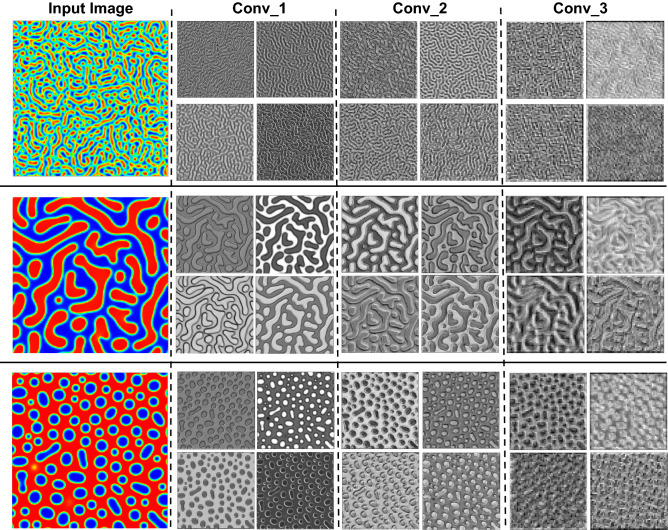
Sample response maps in developed CNN for 2D microstructure morphology inputs. The response map of the first four filters of three convolutional layers is illustrated for three input images. The layer numbers are presented at the top of the images.

**Figure 6:**
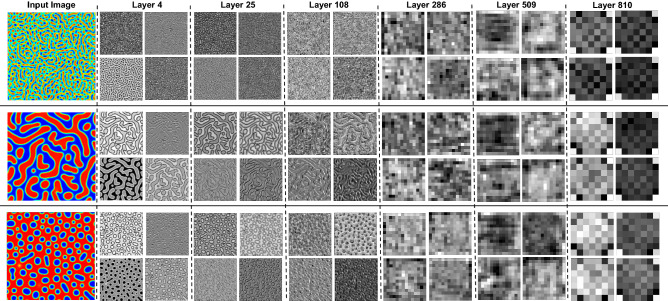
Sample response maps in EfficientNetB7 for 2D microstructure morphology inputs. The response map of the first four filters of some convolutional layers is illustrated for three input images. The layer numbers are presented at the top of the images.

The overreaching goal of the convolutional layers is feature extraction from the images. First, we train a proposed CNN, which includes three convolutional layers, batch normalization, max pooling, and ReLU activation function. Filters in each convolutional layer encode the salient features of images. Once the input images are fed into the network, the filters in the convolutional layers are activated to produce the response maps as an output of the filters. Some response maps of each convolutional layer in the proposed CNN are given in Fig. 5. Then, as a comparison, the EfficientNet-B6 and EfficientNet-B7 convolutional layers were also applied to extract the salient features of produced microstructure by the PF method. The EfficientNet-B6 and EfficientNet-B7 have 43 and 66 million parameters which are less than other network parameters with similar accuracy. The trained weights and biases of the EfficientNet models on the ImageNet dataset for classification tasks are loaded for convolutional layers without top fully-connected layers. EfficientNet-B6 and EfficientNet-B7 have 668 and 815 layers, including 139 and 168 convolutional layers, respectively. The response maps for some layers are given in Fig. 6 and Fig. S2 for EfficientNetB7 and EfficientNetB6, respectively. They represent the locations of the encoded features by the filters on the input image.

The response maps for both trained CNN and pretrained EfficientNet show that the first layers capture the simple features like edges, colors, and orientations, while the deeper layers extract more complicated features that are less visually interpretable, see Fig. 6; similar observations are reported in other studies^53,55,89^. The filters from the first layers can extensively detect the edges; hence the microstructures are segmented by the borders of two different phases. By going into deeper layers, understanding the extracted information by the filters becomes more difficult and can only be analyzed by their effects on the accuracy of the final model. Since the pretrained EfficientNet has deeper layers, they can extract more complicated features from the microstructure morphologies. Indeed, we can use different layers for microstructure information extraction and test them to predict the processing history and find the most optimum network.

### Temperature and chemical compositions prediction

**Figure 7:**
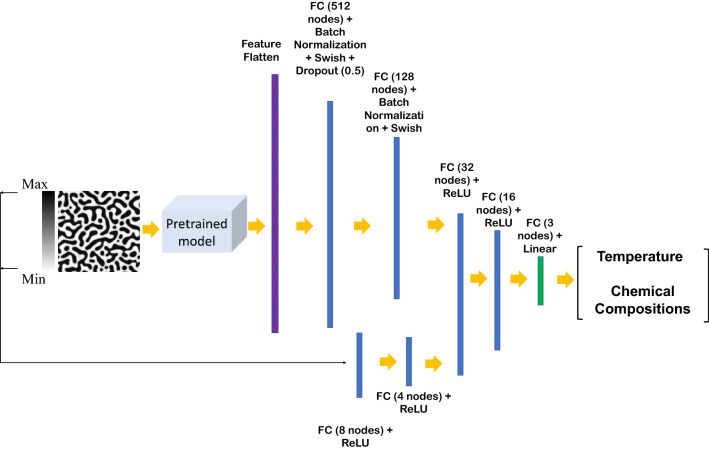
The architecture of the proposed model (input image size is 224 × 224 pixels).

The mixed dataset contains microstructure morphologies as image data and the minimum and maximum of Fe composition in the microstructures as numeric data. The most common reported experimental images in literature for the spinodally decomposed microstructures are greyscale TEM images. To enable the model to predict the chemistry and processing history of the experimental microstructures, we have used the greyscale images in the network training. The proposed CNN, as well as EfficientNet-B6 and EfficientNet-B7 pretrained networks, were used for microstructures’ feature extraction. Then, the extracted features are passed through the fully-connected layers with batch normalization, Swish activation function, and dropout. The numeric data was proceeded by fully-connected layers with the ReLU activation function. The output of both layers was combined with other fully-connected layers to predict temperature and chemical compositions through the linear activation in the last fully-connected layer. After testing different fully-connected layer sizes, the best architecture was selected based on prediction accuracy and stability, which is shown in Fig. S1, supplementary materials, for the proposed CNN and Fig. 7 for pretrained networks. The models were trained on XSEDE resources^90^.

As a starting point, the proposed CNN network with fully-connected layers was trained to predict the processing history parameters. After testing different CNN architectures, the presented network in Fig. S1, in Supplementary Materials, provided the best results that are given in Fig. S3. The results show that the proposed network can predict the chemical compositions reasonably well, but the temperature accuracy is poor. Temperature is a key parameter in the spinodal decomposition process and developing a model with higher accuracy is required. To increase the accuracy, we need to extract more subtle features from the morphologies. However, training a CNN with more layers requires numerous training data. A pretrained network can extract more valuable features from images and consequently can be helpful for accuracy improvement. Therefore, after fixing the architecture of fully-connected layers, different layers of EfficientNet-B6 and EfficientNet-B7 were tested to find the best layer for microstructures’ feature extraction. Herein, layers 96, 111, 142, 231, 304, 319, 362, 392, 496, 556, 631, 659, and 663 from EfficientNet-B6 and layers 25, 108, 212, 286, 346, 406, 464, 509, 613, 673, 806, and 810 from EfficientNet-B7 were selected to quantify the microstructures. The models were run, based on the given parameters in Table 2, for different layers. The model training was repeated five times. The average R Squares and mean square error (MSE) for cross-validation and test set are given in supplementary materials, Tables S2 and S3, for EfficientNet-B6 and EfficientNet-B7, respectively. Indeed, the models were validated by fivefold cross-validation during training, and the test set contains the data that the model never sees in the training process. According to the results, both trained models based on EfficientNet-B6 and EfficientNet-B7 can predict the Co composition very well and while the prediction of temperature and Cr composition is good, they are more challenging. Accordingly, the most accurate prediction belongs to the models that use up to layer 319 of the EfficientNet-B6 and layer 806 of EfficientNet-B7 for microstructures’ quantification.

**Figure 8:**
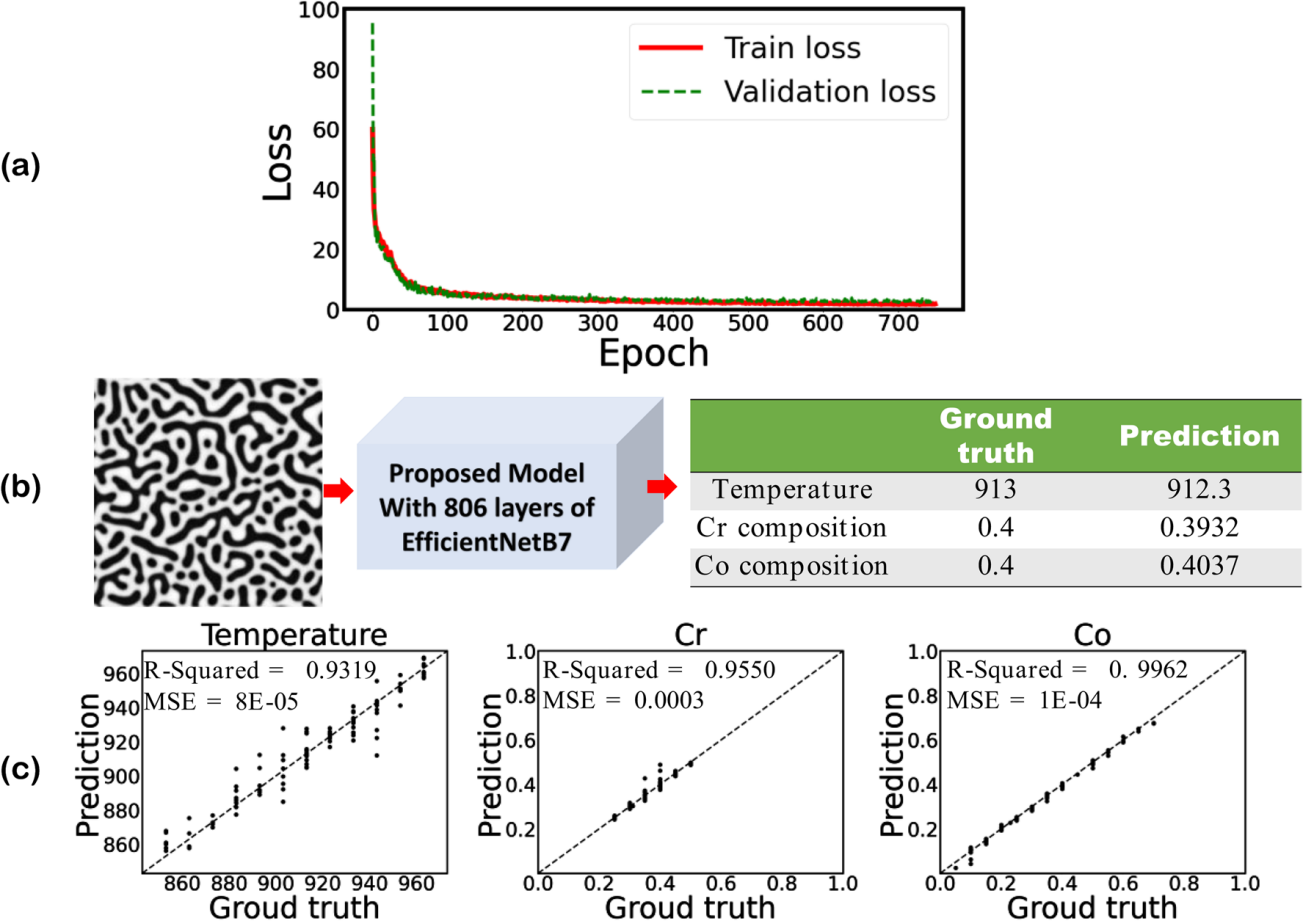
(**a**) Training and validation loss per each epoch, (**b**) prediction of temperature and chemical compositions for a random test dataset, and (**c**) the parity plots of temperature and chemical compositions for the testing dataset from the proposed model when first 806 layers of EfficientNetB7 are used for microstructures’ feature extraction (The size of the input images are 224 × 224 pixels).

In addition to cross-validation and test set accuracy, which can be used for overfitting identification, tracking the loss change in each epoch during the training process can also help in overfitting detection. Figure 8a depicts the loss change in each epoch for the developed model based on EfficientNet-B7, a corresponding plot for EfficientNet-B6 is available in supplementary materials (Fig. S4a). Figure 8a shows that both training and validation losses reduce smoothly with the epoch increase. The insignificant gap between the train and validation losses proves that the models’ parameters converge to the optimal values without overfitting. To better understand the application of the developed models, the models were tested by a sample from the test set; the microstructure belongs to the spinodal decomposition of 20% Fe, 40% Cr, and 40% Co at 913 K after 100 h. The model predictions for temperature and chemical compositions are given in Fig. 8b, for EfficientNet-B7, and Fig. S4b, for EfficientNet-B6. The comparison between the ground truth and prediction demonstrates that the models can predict the chemistry and processing history reasonably well. To quantify the models’ predictive accuracy on all test data points, we have used the parity plots in which the models’ predictions are compared with ground truth in an *x–y* coordinate system. For an ideal 100% accurate model all data points will overlap on a 45-degree line. The parity plots of the models, i.e., EfficientNet-B7 and EfficientNet-B6, for temperature, Cr composition, and Co composition along with their accuracy parameters are given in Fig. 8c and Fig. S4c. The results show that the models can predict the Co composition with the highest accuracy. It seems that temperature prediction is the most challenging variable for the models, but still, there is a good agreement between the models’ prediction and ground truth.

The results include two important points. First, while the extracted features from the shallow trained CNN can predict the compositions well, we need deep CNN to precisely predict the temperature. For this reason, the deep pretrained EfficientNet networks were used, which could predict temperature with higher accuracy. This observation indicates that the compositions are more relevant to simple extracted features of the microstructure morphology, however, more complicated extracted features are required to estimate the temperature. The physical concepts of the problem can also explain this. A small change in compositions would alter the microstructure morphology much more dramatically than a small change in temperature. The differences among the microstructures with different compositions and the same processing temperature are easily recognizable. For example, with a slight change in chemistry the volume fraction of the decomposed phases would vary and this information, i.e., change in the number of white and black pixels, can easily get extracted from the very first layers of the network. However, there are subtle differences between the microstructure morphologies when we slightly change the processing temperature. Therefore, much more complex features are needed to distinguish the differences among the morphologies with small processing temperature variations. Extraction of these complex features requires deeper convolutional layers. In addition, with convolutional layers increasing, the receptive field size would improve. And that ensures no important information is left out from the microstructure when making predictions. Therefore, more information is extracted from the microstructures, and it would also increase the temperature prediction accuracy. On the other hand, training a deep CNN with limited training and test dataset is not practical. To overcome this challenge, transfer learning can be helpful, and some other studies have shown that pretrained networks are effective in feature extraction in materials science-related micrographs^12,23,39,60,91–93^.

## Validation of the proposed model with the experimental data

**Figure 9:**
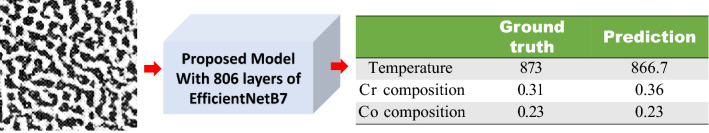
Prediction of chemistry and processing temperature for an experimental TEM image adopted from Okada et al.^94^. The original image was cropped to be in the desired size of 224 × 224 pixels.

The model accuracy against the test dataset, i.e., the data that the model has never seen in the training process, is good, but the test dataset is still from phase-field simulation. Since the ultimate goal of the developed framework is to facilitate the microstructure mediated materials design via predicting chemistry and processing history for experimental microstructures, it is valuable to test the model accuracy on the real microstructures. For this purpose, we have tested the model against an experimental TEM image for spinodal decomposition of Fe–Cr–Co with initial composition 46% Fe, 31% Cr, and 23% Co after 100 h heat treatment at 873 K from Okada et al.^94^. Since the Fe composition of the micrograph was not reported in Okada et al.’s paper, we selected the Fe composition by interpolating between the adjacent simulation points in our database. Figure 9 shows the predictions of the proposed network for an experimental TEM microstructure.

While Co composition and processing temperature prediction is very good, we see a 16% error in Cr composition prediction. We believe the error could stem from several factors. Firstly, the TEM micrograph that we used does not have the image quality of the training dataset. Secondly, the Fe composition associated with the micrograph was not reported in the original paper^94^, and we used a phase-field-informed Fe composition. Thirdly, the dimension of the experimental image was larger than the simulated data, and it was cropped to be at the same size as the required input microstructure size. Despite all these limitations, the proposed model based on the first 806 convolutional layers of EfficientNetB7 predicts the chemistry and processing temperature of an experimental TEM image reasonably well. And it demonstrates that the developed model in this work is suitable for finding the process history behind the experimental microstructures.

Beyond the specific model alloy that we used in this work, the developed model can also be generalized to other materials by considering the material production processes. The developed framework can be used for other ternary alloys that are produced by spinodal decomposition. The model performance in the process history and chemistry prediction should be considered for other spinodal decomposed alloys with less or more elements. The domain adaptation methods such as unsupervised domain adaptation^95^ can provide the ability to use the developed model for other spinodal decomposed alloys. In practice, the proposed model needs two experimental inputs, 1) a TEM micrograph that shows the morphology and, 2) X-ray fluorescence spectroscopy (XRF) that provides the corresponding compositions.

## Conclusion

We introduced a framework based on a deep neural network to predict the chemistry and processing history from the materials’ microstructure morphologies. As a case study, we generated the training and test dataset from phase-field modeling of the spinodal decomposition process of Fe–Cr–Co alloy. We considered a mixed input dataset by combining the image data, the produced microstructure morphologies based on Fe composition, with numeric data, the minimum and maximum of Fe composition in the microstructure. The temperature and chemical compositions were predicted as processing history. We quantified the microstructures by a proposed CNN and different convolutional layers of EfficientNet-B6 and EfficientNet-B7 pretrained networks. Then, the produced features were combined with the output of a fully-connected layer for numeric data processing by other fully-connected layers to predict processing history. After testing different architectures, the best network was found based on the model’s accuracy. A detailed analysis of the model’s performance indicated that the model parameters were optimized based on training and validation loss reduction. The results show that while the simple extracted features from the microstructure morphology by the first convolutional layers are enough for the chemistry prediction, the temperature needs more complicated features that can be extracted by deeper layers. The model benchmark against an experimental TEM micrograph indicates the model’s well predictive accuracy for real alloy systems. We demonstrated that the pretrained convolutional layers of EfficientNet networks could be used to extract the meaningful features relevant to the compositions and temperature from the microstructure morphology. In general, the proposed models were able to predict the processing history based on the materials’ microstructure reasonably well.

## Supplementary Information

Supplementary Information.

## Data Availability

The raw/processed data and codes required to reproduce these findings are available at https://github.com/Amir1361/Materials_Design_by_ML_DL.
