# Supplementary material for: Deep learning approach for chemistry and processing history prediction from materials microstructure
Source: Sci Rep. 2022 Mar 16;12:4552. doi: 10.1038/s41598-022-08484-7 (PMC8927426; doi:10.1038/s41598-022-08484-7)
Supplement: Supplementary file 1 — Supplementary Information. [file 41598_2022_8484_MOESM1_ESM.docx]

**Supplementary Materials**

**Deep Learning Approach for Chemistry and Processing History Prediction from Materials Microstructure: Application to Spinodal Decomposition**

**S.1. Phase-field modeling input parameters**

The coupled Cahn-Hilliard equations were solved by MOOSE’s prebuilt series of weak form residuals of the Cahn-Hilliard equations. The required input parameters are given in Table S1.

Table S1. Phase-field model input parameters ^1-3^

| Gas constant, $R$ $\left[ \frac{J}{K\cdot mol} \right]$ | 8.31446261815324 |
| --- | --- |
| Gradient energy coefficient, $\kappa$ $\left[ \frac{{J\cdot m}^{2}}{mol} \right]$ | 1.0$\times{10}^{14}$ |
| Gibbs free energy of the pure element $i$, $f_{i}^{0}\left[ \frac{J}{mol} \right]$ | $f_{Fe}^{0}=f_{Cr}^{0}=f_{Co}^{0}=0$ |
| Interaction parameters, $L_{ij}$ $\left[ \frac{J}{mol} \right]$ | $L_{Fe,Cr}=$ 20500-9.68$T$  $L_{Fe,Co}=-23669+103.9627T-12.7886T lnT$  $L_{Cr,Co}=$ $\left( 24357-19.797T \right)-2010\left( c_{3}-c_{2} \right)$ |
| Curie temperature, $T_{C}$ $\left[ K \right]$ | $T_{C}=1043c_{Fe}-311.5c_{Cr}+{1450c}_{Co}+\left\{ 1650+550\left( c_{Cr}-c_{Fe} \right) \right\}c_{Fe}c_{Cr}+590c_{Fe}c_{Co}$ |
| Atomic magnetic moment, $\beta$ $\left[ \frac{J}{T} \right]$ | $\beta=2.22c_{Fe}-0.01c_{Cr}+1.35c_{Co}-0.85c_{Fe}c_{Cr}+\left\{ 2.4127+0.2418\left( c_{Co}-c_{Fe} \right) \right\}c_{Fe}c_{Co}$ |
| Magnetic contribution’s $\tau$ function, $f\left( \tau\right)$ $\left[ - \right]$ | $f\left( \tau\right)=\left\{ \begin{matrix} 1-\frac{1}{A}\left[ \frac{79\tau^{-1}}{140p}+\frac{474}{497}\left( \frac{1}{p}-1 \right)\left( \frac{\tau^{3}}{6}+\frac{\tau^{9}}{135}+\frac{\tau^{15}}{600} \right) \right], & \tau<1 \\ -\frac{1}{A}\left( \frac{\tau^{-5}}{10}+\frac{\tau^{-15}}{315}+\frac{\tau^{25}}{1500} \right), & \tau\geq1 \\ A=\frac{518}{1125}+\frac{11692}{15975}\left( \frac{1}{p}-1 \right), & p=0.4 for bcc \end{matrix} \right.$ |
| Elastic coefficients, $C_{ijkl}$ $\left[ GPa \right]$ | $C_{11}^{Fe}=C_{11}^{Co}=233.1$  $C_{12}^{Fe}=C_{12}^{Co}=135.44$  $C_{44}^{Fe}=C_{44}^{Co}=117.83$  $C_{11}^{Cr}=350$  $C_{12}^{Cr}=67.8$  $C_{44}^{Cr}=100.8$ |
| Lattice mismatch, $\varepsilon_{i}$ $\left[ - \right]$ | $\varepsilon_{Cr}=6.1\times{10}^{-3}$  $\varepsilon_{Co}=-7.1\times{10}^{-3}$ |
| Self-diffusion coefficient of element $i$, $D_{i}^{0}$ $\left[ \frac{m^{2}}{s} \right]$ | $D_{Fe}^{0}=D_{Co}^{0}=1.0\times{10}^{-4}$  $D_{Cr}^{0}=2.0\times{10}^{-5}$ |
| Diffusion activation energy of element $i$, $Q_{i}$ $\left[ \frac{kJ}{mol} \right]$ | $Q_{Fe}=Q_{Co}=294$  $Q_{Cr}=308$ |

**S.2. The architecture of the proposed network with developed convolutional layers**

Because we are dealing with a mixed dataset, including both image and numeric data, the proposed CNN was used to extract the features of the microstructures, and the numeric data was proceeded by fully-connected layers with the ReLU activation function. After processing the extracted features through the fully-connected layers with batch normalization, Swish activation function, and dropout, the output of both layers was combined with other fully-connected layers to predict the temperature and chemical compositions through the linear activation in the last fully-connected layer. The architecture of the proposed network is shown in Figure S1.

Figure S1. The architecture of the proposed network with developed convolutional layers (activation='relu', kernel_initializer='he_uniform', padding='same') for microstructures morphology feature extraction (input images size is 224 × 224 pixels)

**S.3. Sample response maps for EfficientNetB6 with 2D microstructure inputs**

The filters can encode the salient features of images in each convolutional layer. The filters in the convolutional layers produce a response map for an input image. Some response maps of different convolutional layers in the pretrained EfficientNet-B6 are given in Figure S2.

Figure S2. Sample response maps in EfficientNetB6 with 2D microstructure inputs. The response map of the first four filters of some convolutional layers is illustrated for three input images. The layer number is presented at the top of the figure.

**S.3. Accuracy of the model with different pretrained convolutional layers of EfficientNet-B6 and EfficientNet-B7**

We compared different convolutional layers of pretrained EfficientNet-B6 and EfficientNet-B7 to find the best layer for microstructures’ feature extraction in the manuscript. Layers 96, 111, 142, 231, 304, 319, 362, 392, 496, 556, 631, 659, and 663 from EfficientNet-B6 and layers 25, 108, 212, 286, 346, 406, 464, 509, 613, 673, 806, and 810 from EfficientNet-B7 were selected to quantify the microstructures. The model training was repeated five times, and the average R Squares and root mean square error (RMSE) for cross-validation and test set are given in Table S2 and Table S3.

Table S2. R-squared and MSE of model predictions for training and testing dataset when different layers of EfficientNet-B6 are used for microstructures’ feature extraction.

| Layer | R squared | | | | | | MSE | | | | | |
| --- | --- | --- | --- | --- | --- | --- | --- | --- | --- | --- | --- | --- |
|  | Validation | | | Test | | | Validation | | | Test | | |
|  | T | C_cr_ | C_co_ | T | C_cr_ | C_co_ | T | C_cr_ | C_co_ | T | C_cr_ | C_co_ |
| **96** | 0.9683 | 0.9930 | 0.9985 | 0.9443 | 0.9048 | 0.9966 | 3E-05 | 3E-05 | 3.7E-05 | 5E-05 | 0.0003 | 6E-05 |
| **111** | 0.9607 | 0.9961 | 0.9984 | 0.9347 | 0.9069 | 0.9958 | 4E-05 | 2E-05 | 3.7E-05 | 6E-05 | 0.0004 | 9E-05 |
| **142** | 0.9739 | 0.9962 | 0.9982 | 0.9503 | 0.9335 | 0.9957 | 3E-05 | 2E-05 | 4.1E-05 | 5E-05 | 0.0003 | 9E-05 |
| **231** | 0.9698 | 0.9944 | 0.9983 | 0.9289 | 0.9022 | 0.9944 | 3E-05 | 3E-05 | 4E-05 | 7E-05 | 0.0004 | 1E-04 |
| **304** | 0.9602 | 0.9948 | 0.9976 | 0.9303 | 0.9328 | 0.9952 | 4E-05 | 3E-05 | 4.8E-05 | 8E-05 | 0.0003 | 7E-05 |
| **319** | 0.9742 | 0.9949 | 0.9987 | 0.9544 | 0.9362 | 0.9964 | 3E-05 | 3E-05 | 3.1E-05 | 5E-05 | 0.0003 | 9E-05 |
| **362** | 0.9626 | 0.9942 | 0.9984 | 0.9324 | 0.9270 | 0.9962 | 4E-05 | 3E-05 | 3.7E-05 | 7E-05 | 0.0003 | 9E-05 |
| **392** | 0.9761 | 0.9939 | 0.9985 | 0.9680 | 0.9151 | 0.9957 | 3E-05 | 3E-05 | 3.5E-05 | 3E-05 | 0.0004 | 9E-05 |
| **496** | 0.9506 | 0.9667 | 0.9962 | 0.8878 | 0.9313 | 0.996233 | 5E-05 | 0.0002 | 8E-05 | 8E-05 | 0.0002 | 7E-05 |
| **556** | 0.9591 | 0.9933 | 0.9977 | 0.9182 | 0.9527 | 0.9962 | 4E-05 | 4E-05 | 5.5E-05 | 9E-05 | 0.0002 | 9E-05 |
| **631** | 0.9198 | 0.9856 | 0.9633 | 0.8143 | 0.8805 | 0.9373 | 7E-05 | 5E-05 | 0.00089 | 0.0002 | 0.0006 | 0.002 |
| **659** | 0.9584 | 0.9925 | 0.9978 | 0.8799 | 0.9313 | 0.9943 | 5E-05 | 4E-05 | 5.1E-05 | 9E-05 | 0.0003 | 1E-04 |
| **663** | 0.9168 | 0.9859 | 0.9607 | 0.8576 | 0.9295 | 0.9222 | 6E-05 | 7E-05 | 0.00096 | 0.0002 | 0.0004 | 0.002 |

Table S3. R-squared and MSE of model predictions for training and testing dataset when different layers of EfficientNet-B7 are used for microstructures’ feature extraction.

| Layer | R squared | | | | | | MSE | | | | | |
| --- | --- | --- | --- | --- | --- | --- | --- | --- | --- | --- | --- | --- |
|  | Validation | | | Test | | | Validation | | | Test | | |
|  | T | C_cr_ | C_co_ | T | C_cr_ | C_co_ | T | C_cr_ | C_co_ | T | C_cr_ | C_co_ |
| **25** | 0.9407 | 0.9906 | 0.9878 | 0.9248 | 0.9112 | 0.9499 | 6E-05 | 5E-05 | 3E-04 | 7E-05 | 0.0003 | 0.001 |
| **108** | 0.9587 | 0.9944 | 0.9988 | 0.9324 | 0.9325 | 0.9956 | 4E-05 | 3E-05 | 3E-05 | 7E-05 | 0.0003 | 1E-04 |
| **212** | 0.9700 | 0.9944 | 0.9982 | 0.9488 | 0.9079 | 0.9935 | 3E-05 | 3E-05 | 4E-05 | 6E-05 | 0.0004 | 1E-04 |
| **286** | 0.9622 | 0.9749 | 0.9980 | 0.9528 | 0.9253 | 0.996 | 4E-05 | 0.0001 | 5E-05 | 5E-05 | 0.0003 | 9E-05 |
| **346** | 0.9687 | 0.9911 | 0.9878 | 0.9451 | 0.8977 | 0.9298 | 3E-05 | 5E-05 | 3E-04 | 6E-05 | 0.0005 | 0.002 |
| **406** | 0.9729 | 0.9959 | 0.998 | 0.9375 | 0.9116 | 0.9964 | 3E-05 | 2E-05 | 5E-05 | 7E-05 | 0.0004 | 9E-05 |
| **464** | 0.9663 | 0.9939 | 0.9983 | 0.9491 | 0.9248 | 0.9972 | 4E-05 | 3E-05 | 4E-05 | 5E-05 | 0.0003 | 8E-05 |
| **509** | 0.9673 | 0.9959 | 0.9984 | 0.9461 | 0.9215 | 0.9952 | 4E-05 | 2E-05 | 4E-05 | 6E-05 | 0.0004 | 1E-04 |
| **613** | 0.9110 | 0.9853 | 0.9696 | 0.8782 | 0.8421 | 0.7446 | 7E-05 | 8E-05 | 7E-04 | 1E-04 | 0.0007 | 0.007 |
| **673** | 0.9625 | 0.9945 | 0.9976 | 0.9484 | 0.9248 | 0.9937 | 4E-05 | 3E-05 | 5E-05 | 6E-05 | 0.0003 | 1E-04 |
| **806** | 0.9599 | 0.9887 | 0.9978 | 0.9319 | 0.955 | 0.9962 | 4E-05 | 6E-05 | 5E-05 | 8E-05 | 0.0003 | 1E-04 |
| **810** | 0.9422 | 0.993 | 0.9976 | 0.9095 | 0.9412 | 0.9963 | 6E-05 | 4E-05 | 6E-05 | 8E-05 | 0.0002 | 1E-04 |

**S.4. The accuracy of proposed networks**

In the manuscript, we discussed the proposed models’ accuracies for the developed CNN and pretrained EfficientNet networks for microstructure morphology features extraction. The loss change in each epoch, the model predictions for temperature and chemical compositions for testing point 20% Fe, 40% Cr, and 40% Co at 913 K after 100 hr, and the parity plots for the models for temperature and chemical compositions along with their accuracy parameters for developed CNN and EfficientNet-B6 are given in Figure S3 and Figure S4, respectively.

Figure S3. a) Training and validation loss per each epoch, b) prediction of temperature and chemical compositions for a test dataset, and c) the parity plots of temperature and chemical compositions for the testing dataset from the proposed model when proposed CNN are used for microstructures’ feature extraction (input image size is 224 × 224 pixels)

Figure S4. a) Training and validation loss per each epoch, b) prediction of temperature and chemical compositions for a test dataset, and c) the parity plots of temperature and chemical compositions for the testing dataset from the proposed model when first 319 layers of EfficientNetB6 are used for microstructures’ feature extraction (input image size is 224 × 224 pixels)

**References**

1 Koyama, T. & Onodera, H. Phase-Field simulation of phase decomposition in Fe− Cr− Co alloy under an external magnetic field. *Metals and Materials International* **10**, 321-326 (2004).

2 Lv, L., Zhen, L., Xu, C. & Sun, X. Phase field simulation of microstructure evolution in Fe–Cr–Co alloy during thermal magnetic treatment and step aging. *Journal of magnetism and magnetic materials* **322**, 987-995 (2010).

3 Hillert, M. & Jarl, M. A model for alloying in ferromagnetic metals. *Calphad* **2**, 227-238 (1978).
